# Supplementary material for: LDLR, LRP1, and Megalin redundantly participate in the uptake of Clostridium novyi alpha-toxin
Source: Commun Biol. 2022 Sep 5;5:906. doi: 10.1038/s42003-022-03873-0 (PMC9445046; doi:10.1038/s42003-022-03873-0)

Supplementary Information

LDLR, LRP1, and Megalin redundantly participate in the uptake of *Clostridium novyi* alpha-toxin

Yao Zhou[1,2,3,4,#], Danyang Li[1,2,3,4,#], Diyin Li[1,2,3,4], Aizhong Chen[2,3,4], Liuqing He[2,3,4], Jianhua Luo[2,3,4] and Liang Tao[1,2,3,4,*]

[1] College of Life Sciences, Zhejiang University, Hangzhou, Zhejiang, 310058, China

[2] Key Laboratory of Structural Biology of Zhejiang Province, School of Life Sciences, Westlake University, Hangzhou, Zhejiang, 310024, China

[3] Center for Infectious Disease Research, Westlake Laboratory of Life Sciences and Biomedicine, Hangzhou, Zhejiang, 310024, China

[4] Institute of Basic Medical Sciences, Westlake Institute for Advanced Study, Hangzhou, Zhejiang, 310024, China

#These authors contributed equally to this work.

*Corresponding to:

Liang Tao, Ph.D. Telephone: +86-571-86929603; Email: taoliang@westlake.edu.cn

This file includes:

Supplementary Figures 1-4

Uncropped original western blots

Supplementary Figures

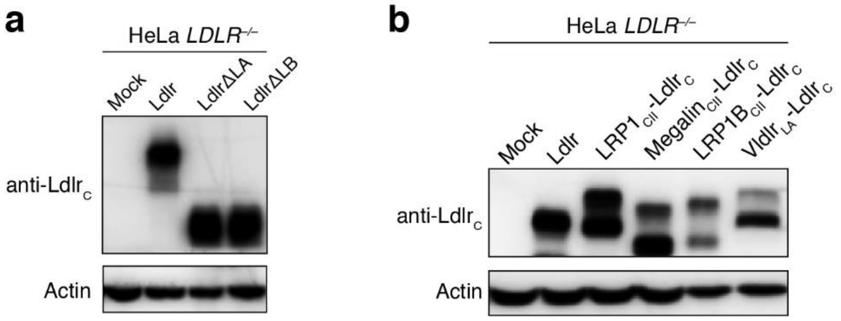

Supplementary Figure 1 │ Expression of truncated/chimeric LDLR family proteins in the HeLa *LDLR*$^{-/-}$ cells.

a The expression of Ldlr, $Ldlr_{\Delta LA}$, and $Ldlr_{\Delta LB}$ in the HeLa *LDLR*$^{-/-}$ cells were shown by immunoblotting using an anti-$Ldlr_C$ antibody. Actin serves as a loading control. **b** The expression of Ldlr, $LRP1_{CII}$-$Ldlr_C$, $Megalin_{CII}$-$Ldlr_C$, $LRP1B_{CII}$-$Ldlr_C$, and $Vldlr_{LA}$-$Ldlr_C$ in the HeLa *LDLR*$^{-/-}$ cells were shown by immunoblotting using an anti-$Ldlr_C$ antibody. Actin serves as a loading control. The experiments have been repeated independently twice with similar results.

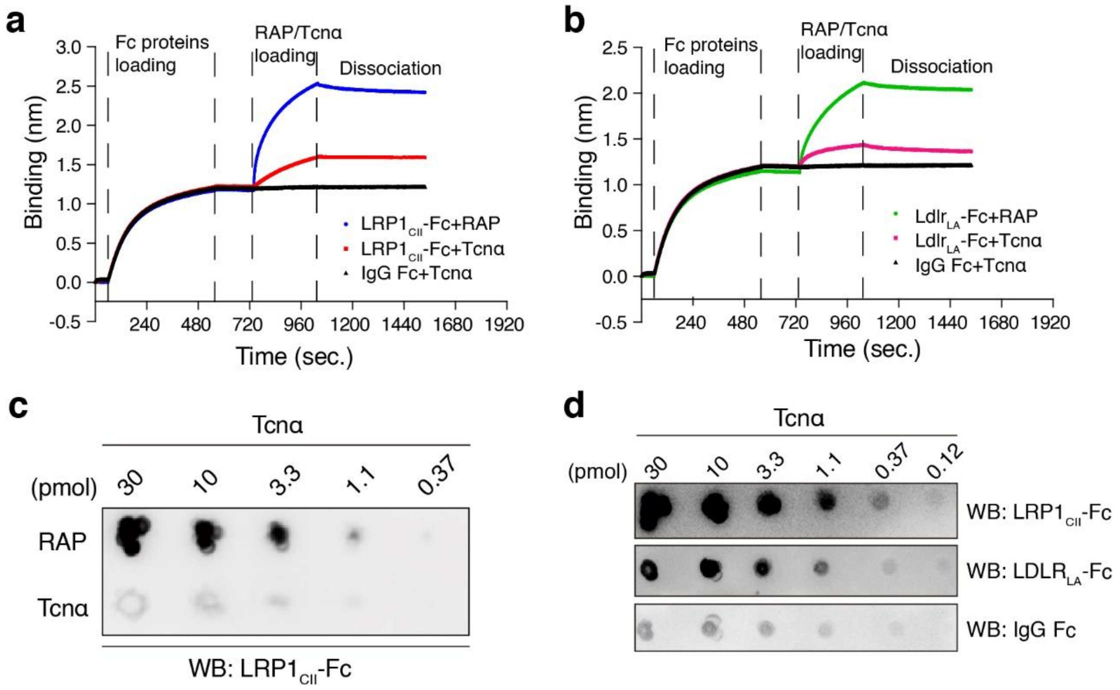

Supplementary Figure 2 │ Direct interactions between Ldlr$_{LA}$ and Tcnα and between LRP1$_{CII}$ and Tcnα are weak.

a Characterization of Tcnα (2.5 μM) binding to immobilized LRP1$_{CII}$-Fc using the BLI assay. RAP (500 nM) serves as a positive control and human IgG Fc serves as a negative control. **b** Characterization of Tcnα (2.5 μM) binding to immobilized Ldlr$_{LA}$-Fc using the BLI assay. RAP (500 nM) serves as a positive control and human IgG Fc serves as a negative control. **c** *In vitro* dot blot assays did not detect binding of LRP1$_{CII}$-Fc to membrane immobilized Tcnα. RAP served as a positive control. **d** *In vitro* dot blot assays with EDC cross-link showed that LRP1$_{CII}$-Fc and LDLR$_{LA}$-Fc, but not IgG Fc, interact with immobilized Tcnα.

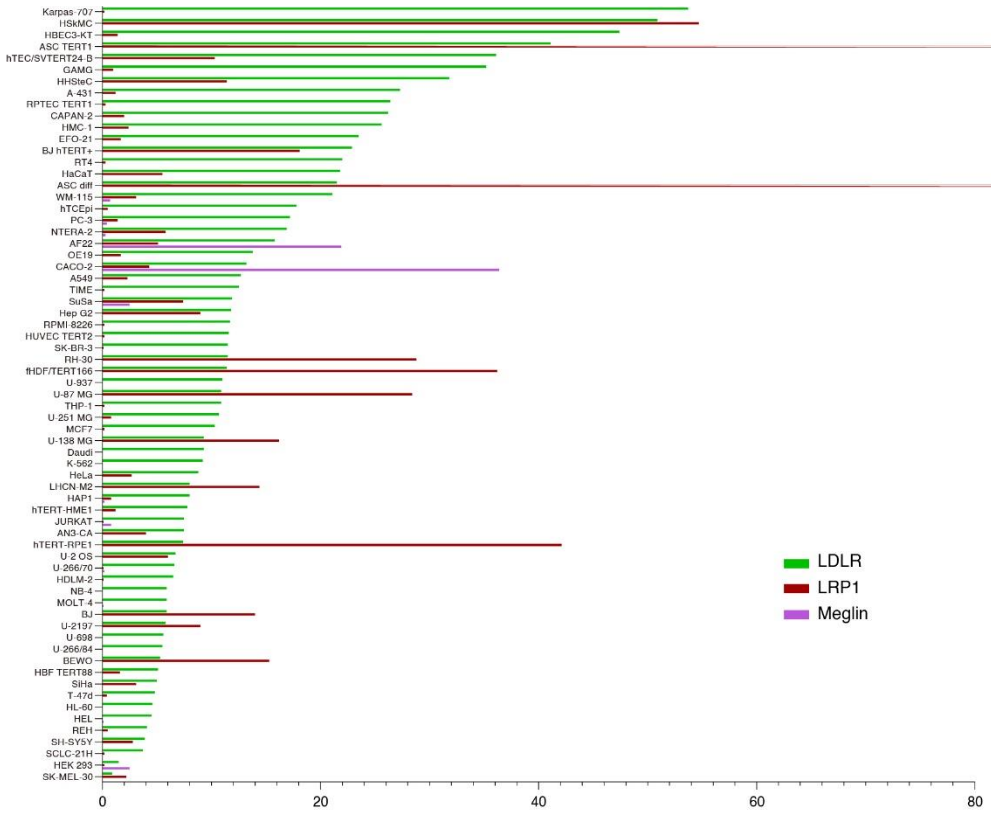

Supplementary Figure 3 │ The comparison of the RNA expression levels of LDLR, LRP1, and Megalin in various cell lines.

Checking and downloading the RNA expression data in a public database (http://www.proteinatlas.org). The rank was based on the mRNA expression level of LDLR from high to low. The data were analyzed by GraphPad Prism.

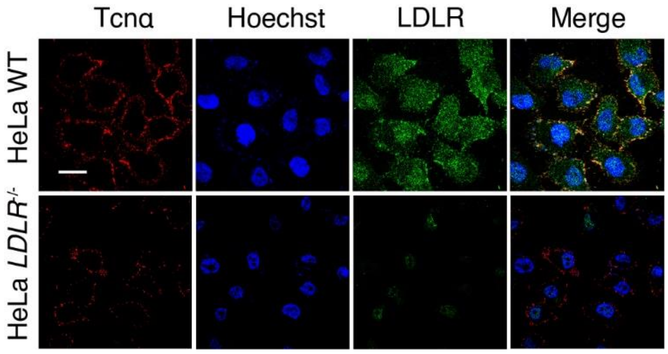

Supplementary Figure 4 │ HeLa *LDLR*$^{-/-}$ cells showed minimal signals for internalized Tcnα. The Tcnα internalization assay was performed with the HeLa WT and *LDLR*$^{-/-}$ cells. The cells were then fixed and subjected to the immunofluorescent assay. Representative images are shown. The scale bar represents 25 μm.

Uncropped original western blots

Figure 4b

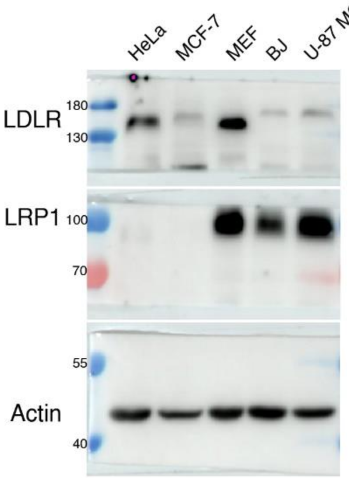

Figure 4c

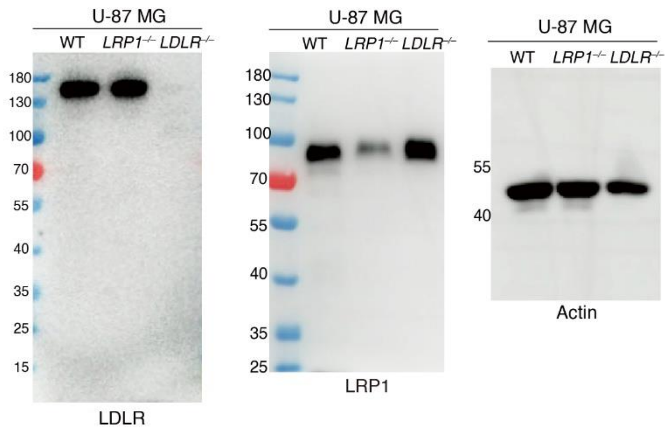

Supplementary Figure 1a

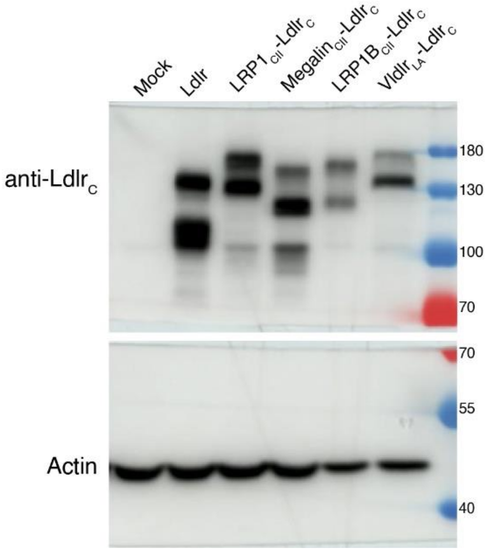

Supplementary Figure 1b

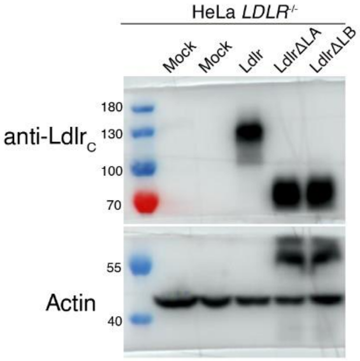

Supplement: Supplementary file 2 — Supplementary Information [file 42003_2022_3873_MOESM2_ESM.pdf]
