# Supplementary material for: LDLR, LRP1, and Megalin redundantly participate in the uptake of Clostridium novyi alpha-toxin
Source: Commun Biol. 2022 Sep 5;5:906. doi: 10.1038/s42003-022-03873-0 (PMC9445046; doi:10.1038/s42003-022-03873-0)
Supplement: Supplementary file 5 — Reporting Summary [file 42003_2022_3873_MOESM5_ESM.pdf]

nature portfolio

Corresponding author(s): Liang Tao

Last updated by author(s): Aug 2, 2022

Reporting Summary

Nature Portfolio wishes to improve the reproducibility of the work that we publish. This form provides structure for consistency and transparency in reporting. For further information on Nature Portfolio policies, see our Editorial Policies and the Editorial Policy Checklist.

Statistics

For all statistical analyses, confirm that the following items are present in the figure legend, table legend, main text, or Methods section.

n/a	Confirmed	
☐	☒	The exact sample size (n) for each experimental group/condition, given as a discrete number and unit of measurement
☐	☒	A statement on whether measurements were taken from distinct samples or whether the same sample was measured repeatedly
☐	☒	The statistical test(s) used AND whether they are one- or two-sided<br>*Only common tests should be described solely by name; describe more complex techniques in the Methods section.*
☒	☐	A description of all covariates tested
☐	☒	A description of any assumptions or corrections, such as tests of normality and adjustment for multiple comparisons
☐	☒	A full description of the statistical parameters including central tendency (e.g. means) or other basic estimates (e.g. regression coefficient) AND variation (e.g. standard deviation) or associated estimates of uncertainty (e.g. confidence intervals)
☐	☒	For null hypothesis testing, the test statistic (e.g. F, t, r) with confidence intervals, effect sizes, degrees of freedom and P value noted<br>*Give P values as exact values whenever suitable.*
☒	☐	For Bayesian analysis, information on the choice of priors and Markov chain Monte Carlo settings
☒	☐	For hierarchical and complex designs, identification of the appropriate level for tests and full reporting of outcomes
☒	☐	Estimates of effect sizes (e.g. Cohen's d, Pearson's r), indicating how they were calculated

Our web collection on statistics for biologists contains articles on many of the points above.

Software and code

Policy information about availability of computer code

Data collection	Olympus IX73 and Zeiss LSM 880 NLO with AiryScan System were used for image data collection.
Data analysis	GraphPad Prism v9.3, OriginPro v8.5, Octet Data Analysis software v12.0.1.2, ImageJ ver1.53

For manuscripts utilizing custom algorithms or software that are central to the research but not yet described in published literature, software must be made available to editors and reviewers. We strongly encourage code deposition in a community repository (e.g. GitHub). See the Nature Portfolio guidelines for submitting code & software for further information.

Data

Policy information about availability of data

All manuscripts must include a data availability statement. This statement should provide the following information, where applicable:

- Accession codes, unique identifiers, or web links for publicly available datasets
- A description of any restrictions on data availability
- For clinical datasets or third party data, please ensure that the statement adheres to our policy

The source data behind the graphs and charts in the paper are provided as Supplementary Data. Uncropped blots are available in Supplementary Information.

Field-specific reporting

Please select the one below that is the best fit for your research. If you are not sure, read the appropriate sections before making your selection.

☒ Life sciences ☐ Behavioural & social sciences ☐ Ecological, evolutionary & environmental sciences

For a reference copy of the document with all sections, see nature.com/documents/nr-reporting-summary-flat.pdf

Life sciences study design

All studies must disclose on these points even when the disclosure is negative.

Sample size	For cell rounding experiments, n=6 for each group.<br>Sample size was determined based on previous report or knowledge. Each sample size was selected so that a reasonable researcher would conclude that the size is sufficient to draw a conclusion. For all experiments, at least two biological replicates were performed.
Data exclusions	No data exclusions.
Replication	All experiments were replicated at least twice. All attempts at replication are successful.
Randomization	Samples were allocated into experimental groups randomly
Blinding	Blinding was not performed as virtually these data are quantitative and would not subject to operator bias easily.

Reporting for specific materials, systems and methods

We require information from authors about some types of materials, experimental systems and methods used in many studies. Here, indicate whether each material, system or method listed is relevant to your study. If you are not sure if a list item applies to your research, read the appropriate section before selecting a response.

Materials & experimental systems

n/a	Involved in the study	
☐	☒	Antibodies
☐	☒	Eukaryotic cell lines
☒	☐	Palaeontology and archaeology
☒	☐	Animals and other organisms
☒	☐	Human research participants
☒	☐	Clinical data
☒	☐	Dual use research of concern

Methods

n/a	Involved in the study	
☒	☐	ChIP-seq
☒	☐	Flow cytometry
☒	☐	MRI-based neuroimaging

Antibodies

Antibodies used	Alexa Fluor 488 goat anti-rabbit IgG (ab150077, 1:1000, Abcam)<br>rabbit polyclonal IgG against β-Actin (ab227387, 1:5000, Abcam)<br>rabbit monoclonal IgG against LDLR (ab52818 for Western blot, 1:500; ab30532 for immunofluorescence, 1:200; Abcam)<br>rabbit monoclonal IgG against LRP1(ab92544, 1:2000 for western blot and 1:200 for immunofluorescence, Abcam)<br>HRP-conjugated goat anti-human IgG-Fc antibody (SSA001, 1:3000, Sino Biological).
Validation	All primary antibodies were validated based on either previous literatures or vendor's instructions.

Eukaryotic cell lines

Policy information about cell lines

Cell line source(s)	HeLa (H1, CRL-1958) and MCF-7 (HTB-22) cells were originally obtained from ATCC. MEFs (CTCC-003-0036), BJ (CTCC-400-0144), and U-87 MG (CTCC-ZHYC-0434) cells were purchased from Chinese Tissue Culture Collections (CTCC). Expi293F cells (A14527) were purchased from ThermoFisher Scientific.
Authentication	HeLa cells were authenticated via STR profiling (Shanghai Biowing Biotechnology Co. LTD, Shanghai, China). Other cells were not authenticated.
Mycoplasma contamination	They were tested negative for mycoplasma contamination.

Commonly misidentified lines (See ICLAC register)	No commonly misidentified lines were used in this study.
